# Supplementary material for: A phyB-PIF1-SPA1 kinase regulatory complex promotes photomorphogenesis in Arabidopsis
Source: Nat Commun. 2019 Sep 16;10:4216. doi: 10.1038/s41467-019-12110-y (PMC6746701; doi:10.1038/s41467-019-12110-y)
Supplement: Supplementary file 1 — Supplementary Information [file 41467_2019_12110_MOESM1_ESM.pdf]

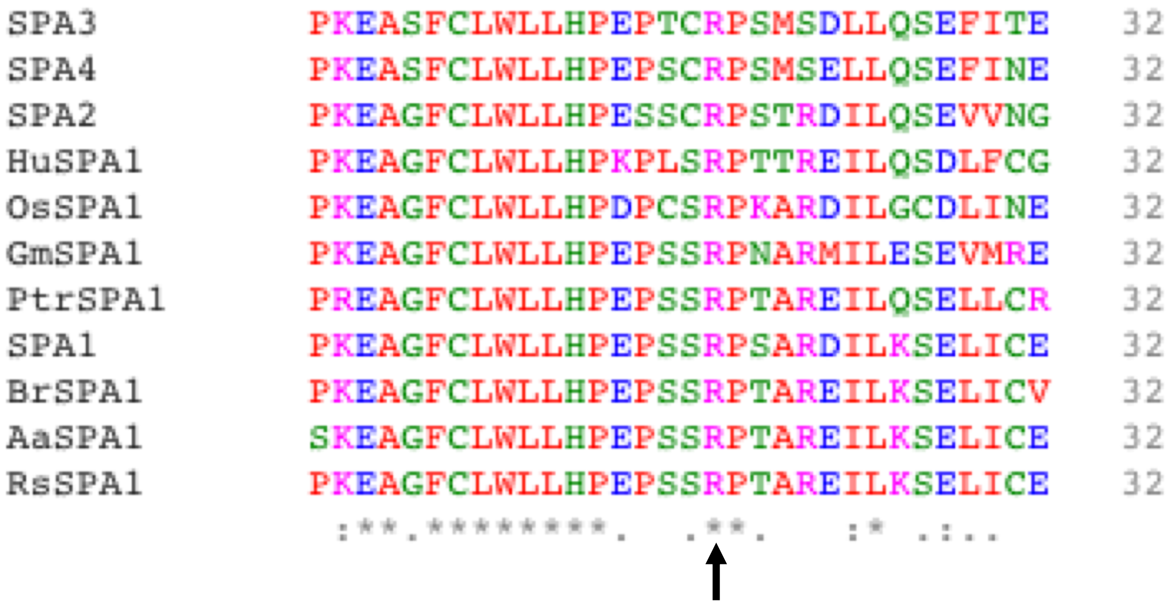

Supplementary Figure 1: Alignment of the kinase domain of four Arabidopsis SPA proteins along with various SPA1 orthologues display a highly conserved arginine residue for the salt-bridge formation. *Os, Oryza sativa; Gm, Glycine max; Aa, Arabis alpine; Br, Brassica rapa; Ptr, Populus trichocarpa; Hu, Herrania umbratical; Rs, Raphanus sativus*.

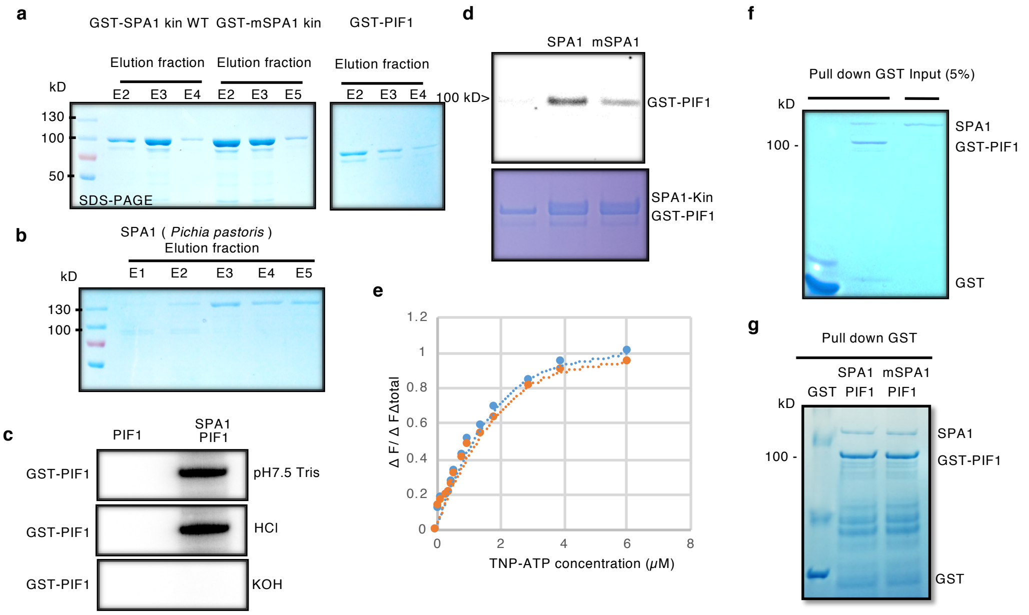

Supplementary Figure 2: Preparation of SPA1 protein and biochemical analysis of SPA1.

(a) Coomassie staining of *E. coli* purified SPA1 kinase domain and mutant protein. N-terminal GST and C-terminal strep tagged wild type and mutant versions of SPA1-Kin (left panel, ~100kDa) were purified using Strep-Tactin Sepharose gravity columns. Total twenty five μl of three different elutions were ran on an 8% SDS-PAGE gel and visualized with Coomassie blue staining. The strep-tagged GST-PIF1 (right panel, ~95kDa) was also purified using the same method and visualized with Coomassie blue staining. (b) Commassie staining of *Pichia pastoris* purified SPA1 (~135KDa) full-length protein. The full-length SPA1-strep was expressed in *Pichia pastoris* and purified using a Strep-Tactin Sepharose gravity column. Five elutions were ran on an 8% SDS-PAGE gel and visualized with Coomassie blue staining. (c) PIF1 phosphorylation by SPA1 is Ser/Thr phosphorylation. A kinase assay was performed with SPA1 and PIF1 in the presence of ɣ32P-labeled ATP. The reactions were divided into three lanes on an 8% SDS-PAGE gel and transferred to PVDF membrane. Each bands were excised and exposed to either HCl or KOH. PIF1 phosphorylation was specifically removed by KOH treatment, indicating that the phosphorylation is on the serine/threonine residues of PIF1. (d) SPA1 kinase domain can phosphorylate PIF1 *in vitro*, whereas mutant SPA1 kinase domain shows reduced phosphorylation of PIF1. *E. coli* purified SPA1-Kin and PIF1 was used in the kinase assay. Autoradiogram shows PIF1 phosphorylation in the presence of SPA1-Kin. The phosphorylation was reduced with mSPA1-Kin. (e) TNP-ATP binding assay showed similar ATP binding to both SPA1 and mutant SPA1 kinase domain. Blue dots indicate wild type SPA1-Kin, whereas red dots indicate mutant mSPA1-Kin. Y axis indicates the value of fluorescence change divided by the saturated total fluorescence change, Δ F/ Δ F total. X axis indicates the concentration of TNP-ATP added to the reaction. (f) SPA1 can specifically interact with PIF1 *in vitro*. GST-PIF1 was used to pull down SPA1-strep protein *in vitro*. GST alone was used as a negative control. Proteins were visualized with Coomassie staining after the pull-down assay. (g) GST pull-down also showed that the wild type and mutant version of SPA1 can equally interact with GST-PIF1 *in vitro*.

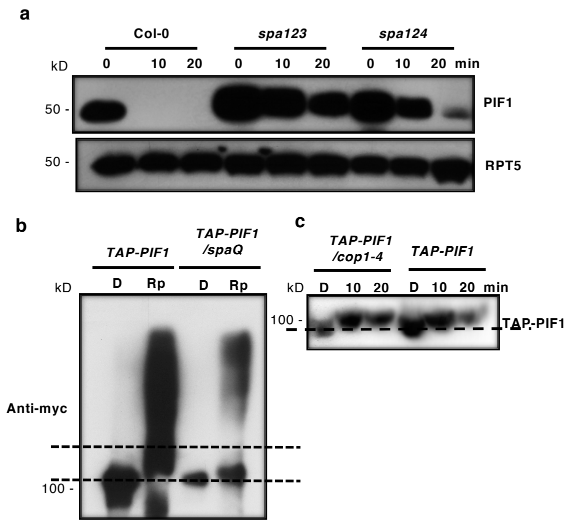

Supplementary Figure 3 : SPAs redundantly function in light-induced PIF1 ubiquitination and degradation *in vivo.*

(a) Red light-induced PIF1-degradation was delayed in *spa123* and *spa124* mutants compared to wild type. Seedlings were grown on the MS plate in the dark for four days before exposing to red light (300 μmolm^{-2}) for the duration indicated in each figure. Seedlings were ground and total proteins were solubilized with the denaturing buffer (100mM Tris-Cl, 8M Urea, 1X Protease inhibitor). PIF1 levels are shown with anti-PIF1 antibody and RPT5 was used as a loading control. (b) PIF1 phosphorylation and subsequent polyubiquitylation is defective in *spaQ* mutant. To examine ubiquitination in *spaQ* mutant, dark-grown seedlings were pre-treated with 40 μM of Bortezomib to inhibit the 26S proteasome activity. After 4 hours of pretreatment, seedlings were exposed to saturated red light (3000 μmolm^{-2}). TAP-PIF1 was immunoprecipitated with anti-myc antibody and ran on a 6.5% SDS-PAGE gel to visualize with Western Blot using anti-myc antibody. (c) COP1 does not affect PIF1 phosphorylation *in vivo*. In *cop1-4* mutant, TAP-PIF1 shows a similar level of phosphorylation with wild type in response to red light. Total proteins were separated on a 6.5% SDS-PAGE gel and blotted with anti-myc antibody. TAP-PIF1 showed similar band shift both in wild type and *cop1-4* mutant.

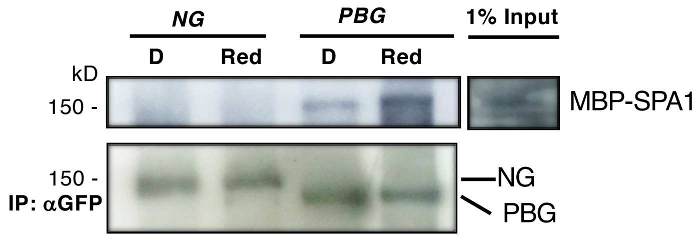

Supplementary Figure 4 : The N-terminal domain of phyB does not interact with SPA1.

Semi- *in vivo* co-immunoprecipitation assay showing red light-induced interaction between PBG and MBP-SPA1. NG lacks SPA1 interaction under the same condition, suggesting the importance of the C-terminal half of phyB for SPA1 interaction.

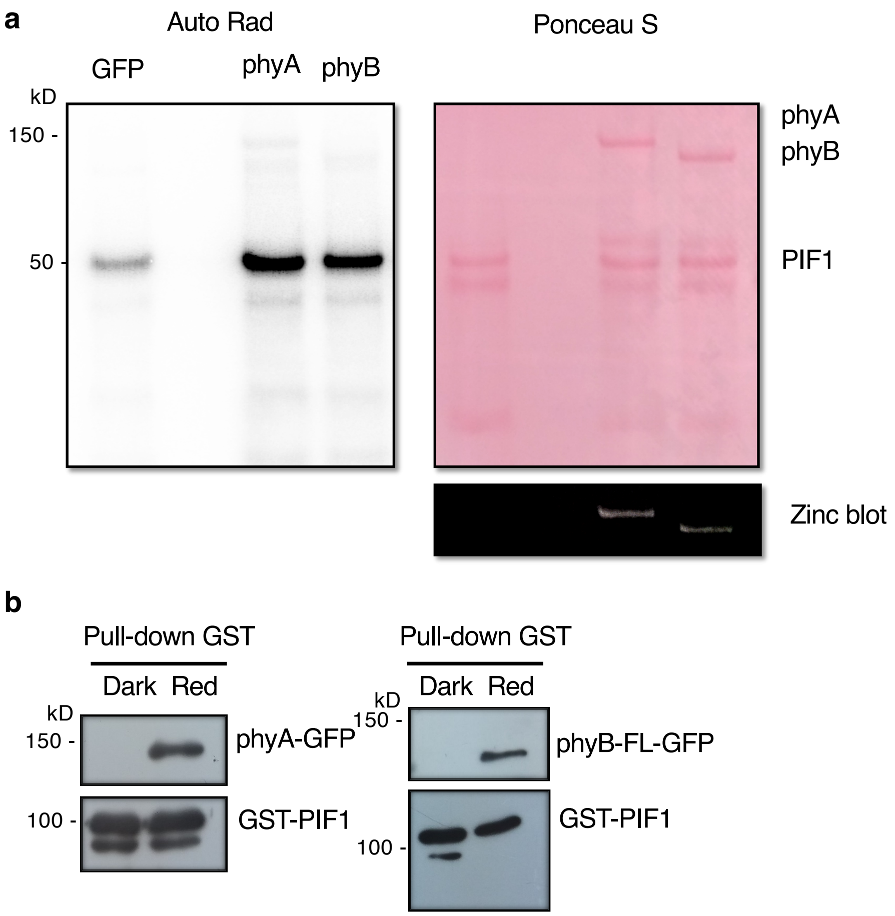

Supplementary Figure 5 : Arabidopsis phyA and phyB can phosphorylate PIF1 *in vitro*.

(a) Purified phyA-GFP and phyB-GFP from *Saccharomyces cerevisiae* showed kinase activity toward PIF1-his *in vitro*. Autoradiogram (left panel) shows phosphorylation of PIF1-his with phyA-GFP an phyB-GFP. GFP alone expressed from the same host, shows background level of the PIF1-his phosphorylation. phyA-GFP and phyB-GFP holoproteins were purified as described in the method section. Kinase assay was performed and total proteins were ran on the 6.5% SDS-PAGE gel. The gel was transferred to PVDF membrane and incubated with Ponceau S staining solution to visualize proteins (right upper panel). The membrane was further incubated with zinc chloride solution and the holoprotein formation with chromophore attachment was shown under UV light (right bottom panel). (b) The integrity of the purified phyA (left panel) and phyB (right panel) has been shown by the *in vitro* light-dependent interaction with PIF1.

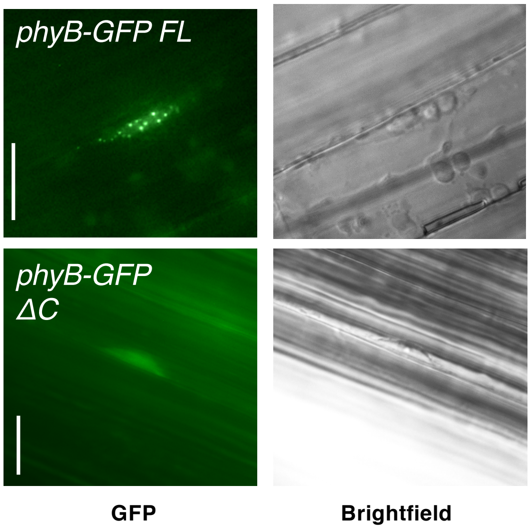

Supplementary Figure 6 : Prolonged red light treatment (16hr) failed to produce phyB photobody formation in *phyB-GFP ΔC* seedlings. The full-length phyB-GFP produces photobodies after 16 hours of red light (10 $\mu molm^{-2}s^{-1}$) treatment whereas phyB-GFP ΔC didn't produce any photobodies under these conditions. Bar = 15 μm.

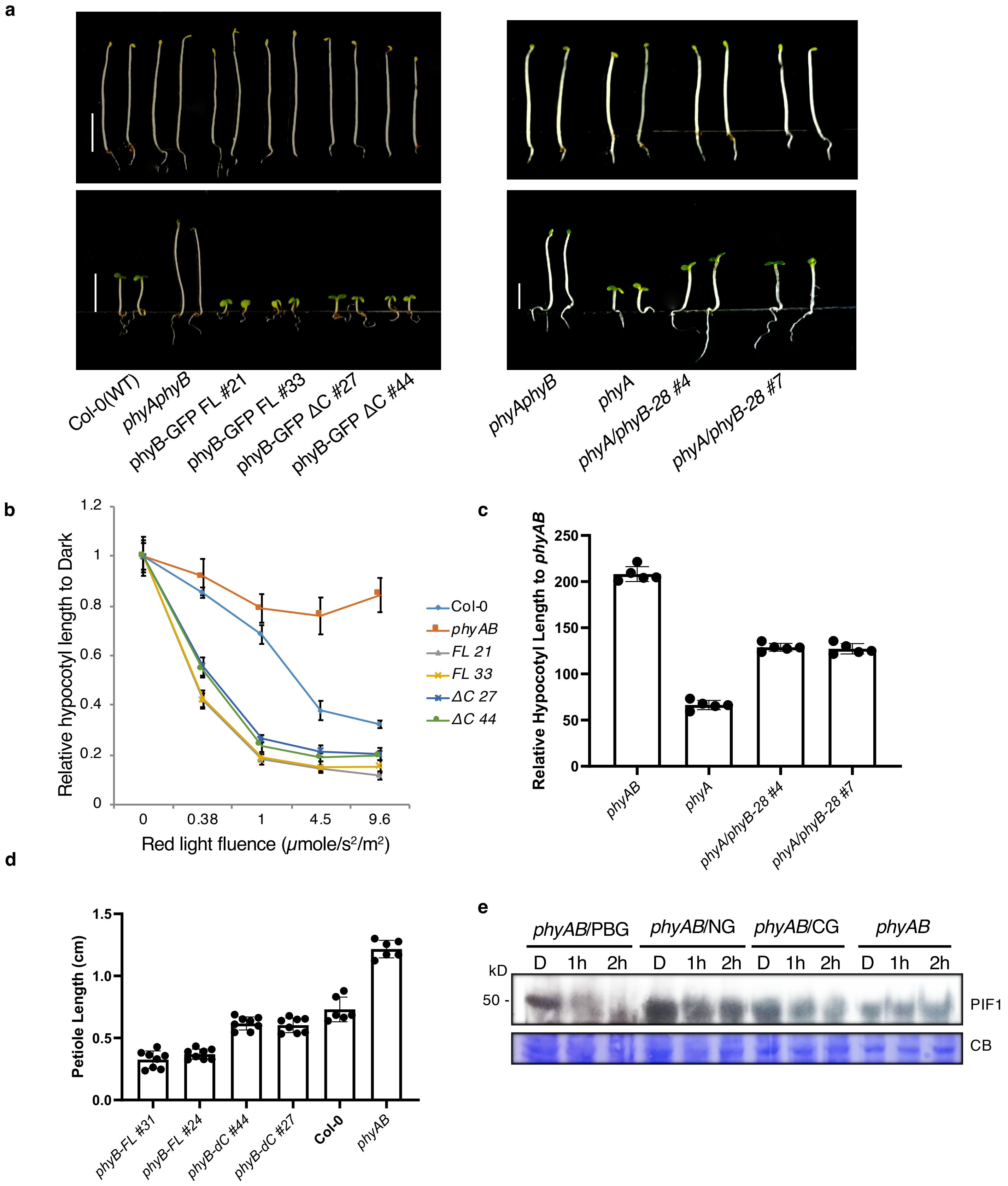

a
Col-0(WT)
phyAphyB
phyB-GFP FL #21
phyB-GFP FL #33
phyB-GFP ΔC #27
phyB-GFP ΔC #44
phyAphyB
phyA
phyA/phyB-28 #4
phyA/phyB-28 #7
b
Relative hypocotyl length to Dark
1.2
1
0.8
0.6
0.4
0.2
0
0.38
1
4.5
9.6
Red light fluence (μmole/s²/m²)
Col-0
phyAB
FL 21
FL 33
ΔC 27
ΔC 44
c
Relative Hypocotyl Length to phyAB
250
200
150
100
50
0
phyAB
phyA
phyA/phyB-28 #4
phyA/phyB-28 #7
d
Petiole Length (cm)
1.5
1.0
0.5
0.0
phyB-FL #31
phyB-FL #24
phyB-dC #44
phyB-dC #27
Col-0
phyAB
e
phyAB/PBG
phyAB/NG
phyAB/CG
phyAB
D
1h
2h
kD
50 -
PIF1
CB

Supplementary Figure 7 : The *phyB-GFP ΔC* and *phyB-28* display multiple phytochrome related phenotypes.

(a) Hypocotyl lengths of *phyB-GFP FL*, *phyB-GFP ΔC* and *phyB-28*. The C-terminal deletion resulted in longer hypocotyls and hyposensitive phyB responses compared to their corresponding wild types in continuous red light (4.5 μmolm^{-2}s^{-1}). Seedlings were grown in the red/ dark for four days before measuring the hypocotyl length using imageJ. Bar = 5mm. (b) Fluence response curves showing hypocotyl lengths in response to various intensities of continuous red light. The hypocotyl lengths of *phyB-GFP ΔC* showed slightly, but significant difference from *phyB-GFP FL* under a wide range of red light conditions. (c) Bar graph showing hypocotyl lengths of *phyB-28* mutant version of *PHYB* transformed into the *phyAB* mutant background under 4.5 μmolm^{-2}s^{-1} of continuous red light. *phyA-211* mutant that has the wild type copy of *PHYB* and was used as a control. (d) *phyB-GFP ΔC* exhibits longer petiole length in 28 day-old white-light-grown plants. (e) PIF1 level in previously reported *PBG* (phyB-GFP), *NG* (phyB N terminal half-GFP-GUS-NLS), and *CG* (phyB C terminal half-GFP) seedlings. Three genotypes of seedlings were grown in dark for four days before transferring to continuous red light (4.5 μmolm^{-2}s^{-1}) for up to two hours. Native PIF1 blot showed significantly delayed degradation of PIF1 in *NG* compared to *PBG*, suggesting the importance of the C-terminal SPA1-interaction domain of phyB in PIF1 degradation.

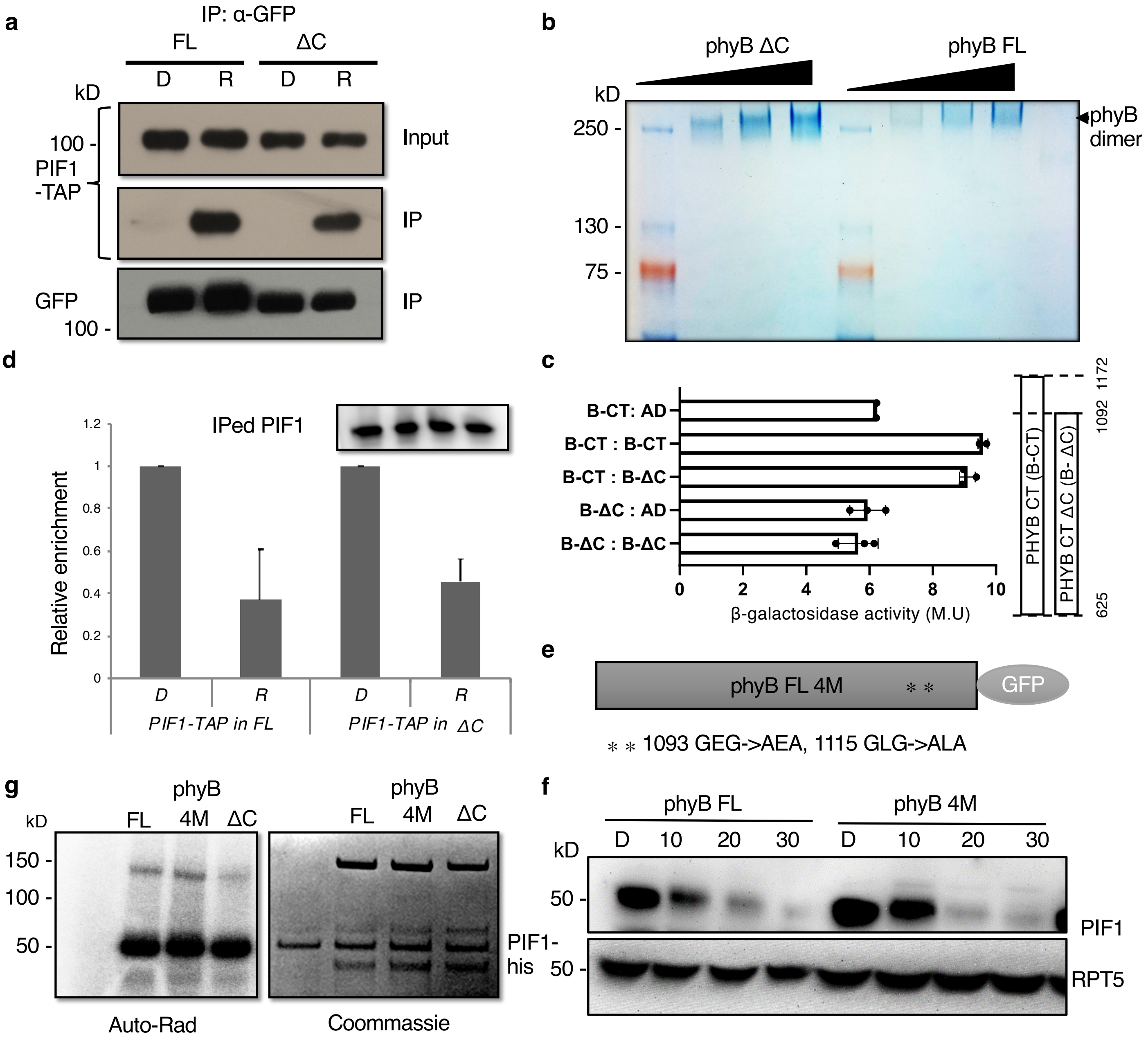

Supplementary Figure 8 : The phyB-GFP ΔC is not defective in other phytochrome functions.

(a) phyB-GFP ΔC can interact with PIF1 in a light-dependent manner similar to phyB-GFP FL *in vivo*. Seedlings were grown in dark for 4 days and the dark-grown seedlings were exposed to a pulse of red light (300 μmolm^{-2}) before sample preparation. Anti-GFP antibody was used to immunoprecipitate phyB-GFP FL and phyB-GFP ΔC. Co-immunoprecipitated proteins were ran into a 6.5% SDS-PAGE gel and blotted onto PVDF membrane. Anti-myc antibody was used to show co-precipitated TAP-PIF1. (b) Both phyB-GFP FL and phyB-GFP ΔC can dimerize *in vitro*. Purified phyB-GFP FL and phyB-GFP ΔC from *Pichia patoris* were ran on a native 6% PAGE gel. The phyB dimers are shown with Coomassie staining. (c) PHYB CT ΔC is unable to dimerize with itself, but can dimerize with PHYB CT domain in yeast two-hybrid assays. (d) Both phyB-GFP FL and phyB-GFP ΔC can sequester PIF1 *in vivo* from the G-box region on the *PIL1* promoter. ChIP assay was performed from seedlings grown either in the dark or dark-grown seedlings exposed to to a pulse of red light (30 μmolm^{-2}). The results showed a similar light-induced reduction of PIF1 occupancy on the *PIL1* promoter between phyB-GFP FL and phyB-GFP ΔC. (e) Schematic structure of phyB-4M. Asterisks indicate the position of the putative G1 and G2 block in the HKRD. (f) PIF1 levels were examined in the phyB-GFP FL and phyB-GFP 4M using native PIF1 antibody. The point mutations on the predicted putative G1 and G2 blocks in HKRD (phyB-GFP 4M) does not alter PIF1 degradation, potentially excluding putative kinase related role of HKRD on PIF1 phosphorylation and degradation. (g) *In vitro* kinase assay shows similar kinase activity of various phyB proteins on PIF1-his. The full-length phyB, 4M and ΔC holoproteins were purified from *Saccharomyces cerevisiae* as describe in the method section. Kinase assay was performed and blotted on the PVDF membrane. PIF1 showed similar phosphorylation by all three different phyB proteins.

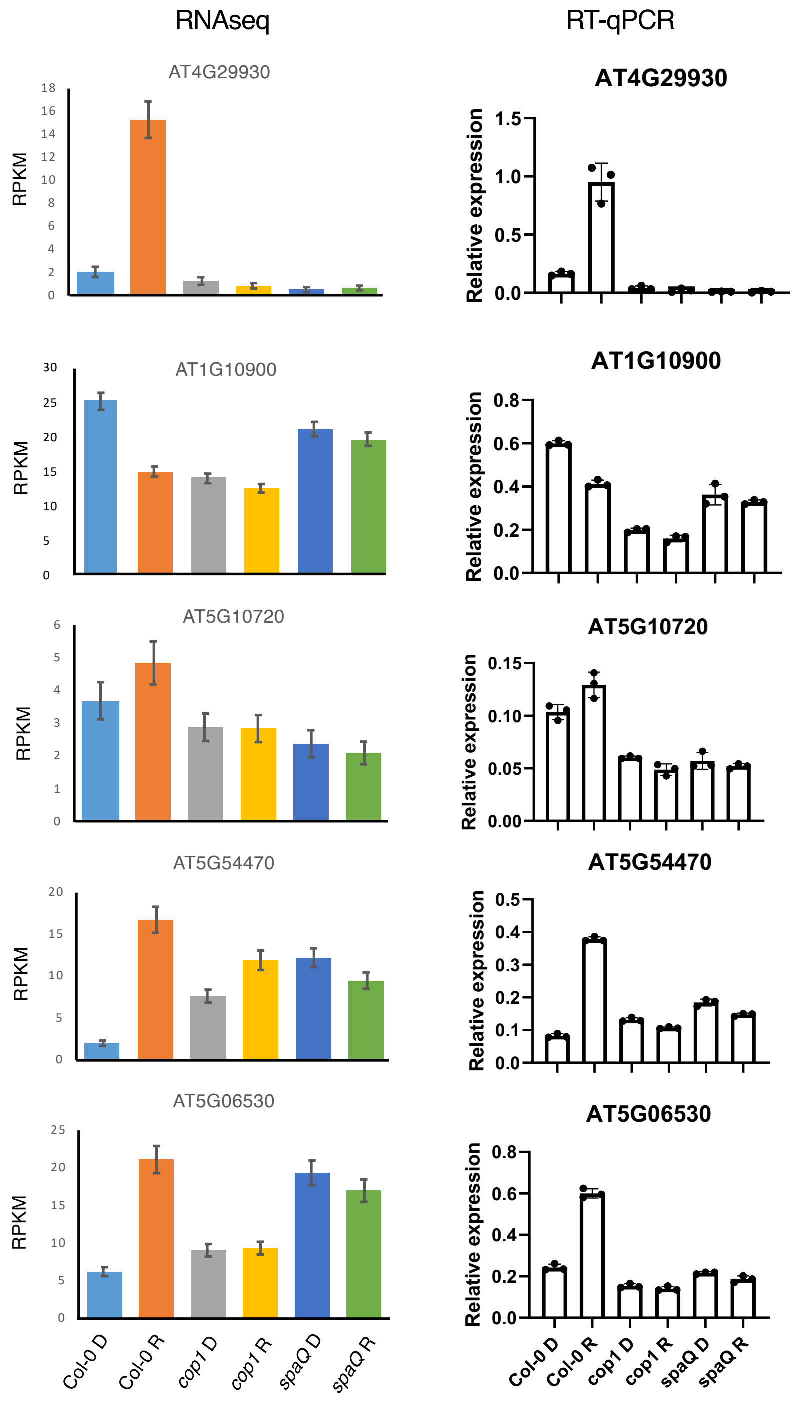

Supplementary Figure 9 : RNA-seq data showed mis-regulation of red-responsive PIF-target genes in *spaQ* and *cop1* mutant.

(Left panel) Three-day-old seedlings of Col-0, *cop1-4* and *spaQ* are either kept in dark or transferred to continuous red light (3.5 μmolm^{-2}s^{-1}) for 1 hour before harvesting the samples. Total RNA was extracted and sequenced as describe in the methods section. Reads Per Kilobase of transcript per Million (RPKM) values were calculated as described in the method section. (**Right panel**) For quantitative RT-PCR, 2 μg of total RNA was reverse transcribed and ran for qPCR using primers listed in supplementary table S1. *PP2A* was used as a control. PIFs target genes that are not red light responsive in *spaQ* and *cop1* are shown. The RPKM values (left panels) were validated by quantitative RT-PCR (right panels). Multiple genes that change expression in response to red light have been affected in *cop1-4* and *spaQ* mutant, suggesting a role of COP1 and SPAs in early red light (1h)-mediated gene regulation. For example, the expression of AT4G29930 (uppermost panels) is significantly induced by red light treatment in wild type, Col-0, whereas the induction was completely abolished in *cop1-4* and *spaQ* mutant.

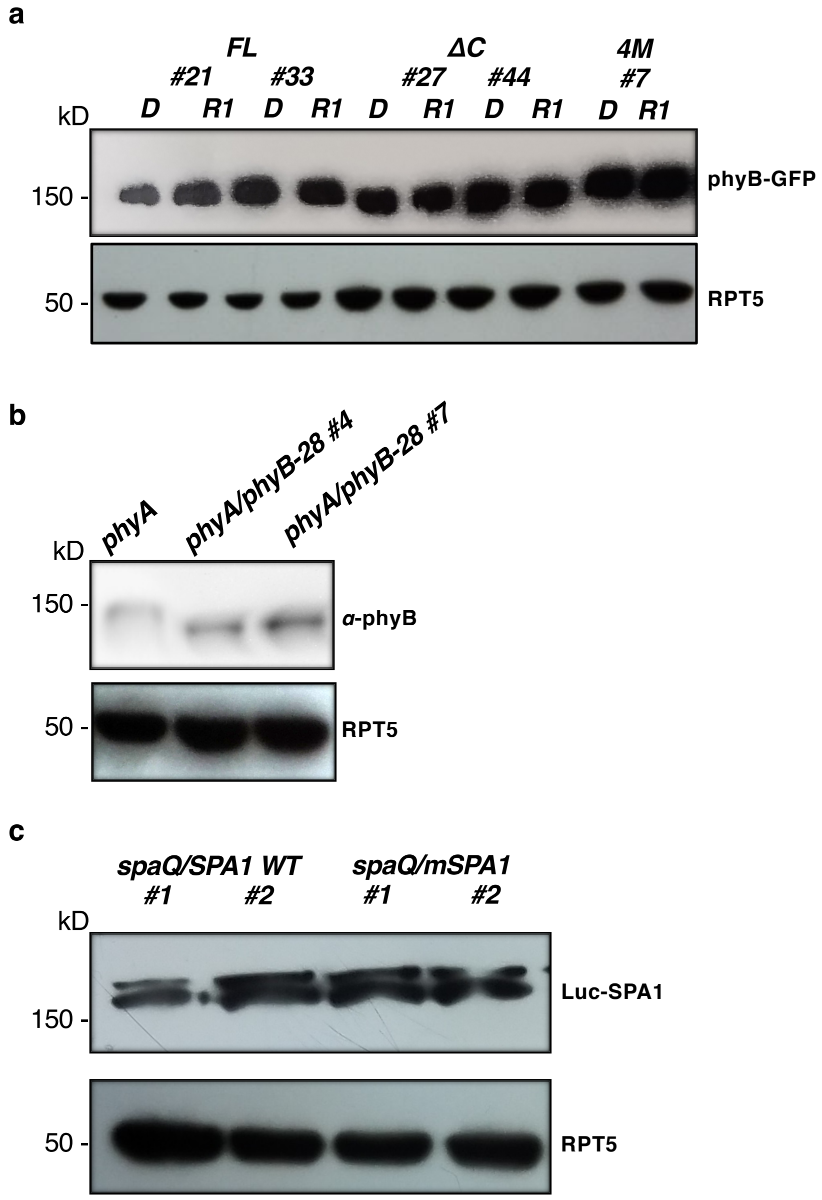

Supplementary Figure 10 : Expression levels of of various proteins in transgenic plants used in this study.

Transgenic plants expressing comparable levels of various proteins were selected and further analyzed in this study. For Western blot analyses, the seedlings of each genotypes were grown in the dark for four days and harvested. For light treated samples (upper panel) continuous red light (3.5 μmolm^{-2}s^{-1}) was irradiated for one hour. Total proteins were solubilized in the same amount of denaturing extraction buffer (100mM Tris-Cl pH 7.5, 8M Urea, 1X Protease inhibitor cocktail) and separated on a 6.5% SDS-PAGE gel. The proteins were blotted on the PVDF membrane and detected with anti-GFP (a), anti-phyB (b) and anti-luciferase (c). RPT5 was used as a loading control.

Supplementary Table 1: Primers and transgenic plants used in this study

Primers	Sequence 5'-3'
**For cloning**	
phyB F KpnI	GAGAGGTACCATGGTTTCCGGAGTCGGGG
phyB FL R SmaI	GTCCCCCGGGCCATATGGCATCATCAGCATCATGTC
phyB ΔC R SmaI	GTCCCCCGGGCCTGGACACGCCATTCTGAATT
phyB 28 R	AAACCCGGG ACCGTCTTCAATGCTTTCAAGATC
SPA1 F BamHI	CCCAGGATCCATGCCTGTTATGGAAAGAGTAG
SPA1 R XhoI	AGAGCTCGAGCTAGTGGTGATGGTGATGATGAACAAGTTTTAGTAGCTTC
SPA1 R kin SalI	CCCAGTCGACCAGCAGCAGTCGATTTAACTG
Strep R NotI	AAAAGCGGCCGCTTAACCACCGAACTGCGGGTG AC
SPA1 R517E F	CCTGAACCCTCCTCCGAACCATCAGCT AGAGATA
SPA1 R517E R	TATCTCTAGCTGATGGTTCGGAGGAGGGTTCAGG
SPA1 F SnaBI	GAGATACGTAGCCACCATGGCTGTTATGGAAAGAGTAG
Flag F SnaBI	GAGATACGTAGGCACCATG GATTACAAGGATGACGAC
Flag R SnaBI	GAGATACGTA GGTACCCTTATCGTCGTCATCC
GFP R XbaI	GAGATCTAGACTACTTGTACAGCTCGTCCATGC
**For ChIP assay**	
PIL1 ChIP F G-box	ATGAATCACGCGGCATTC
PIL1 ChIP R G-box	ACGTGAGCGGAAAGAACC
PIL1 ChIP 3'UTR F	GATGTTTTCTCCAACTAAC
PIL1 ChIP 3'UTR R	AAAATAGTTGGAATTAAGT
**For RT-qPCR**	
AT4G29930 F	CATCTTTGTGTGTCCGAAAAATGGA
AT4G29930 R	TGACCGGAGAGCAAAAAGTCTCTG
AT1G20900 F	GAAACGGCACCGTATCTAACGTCAC
AT1G20900 R	AGTCCCCGTTAGCGAAAGAATCTCA
AT5G10720 F	AGCGCTGATCATGCCCGAAAATAC
AT5G10720 R	CCGGCTTGTCACAGTGAGTTGG
AT5G54470 F	TGGGGAAGAAGAAGTGCGAGTTATG
AT5G54470 R	TGTGTTTCGCCACCAGAAAATTAGC
AT5G06530 F	CGGCGACACGAATACTTCAGTTG
AT5G06530 R	CGCTGTGAAAACAGGGAAGAATCC
**For Y2H assay**	
phyB-F-BamHI	GCTggatcc GTATGAACTCTAAAGTTGTGGAT
phyB-R-SalI	CTGgtcgacTAATATGGCATCATCAGCAT
phyB ΔC-R-SalI	CTGgtcgacTATGGACACGCCATTCTGAATTC

Mutants and Transgenic plants	Source
**Arabidopsis thaliana accession: Col-0**	**Lab stock**
cop1-4	(McNellis et al., 1994)
spaQ	(Laubinger et al., 2004)
TAP-PIF1	(Shen et al., 2008)
TAP-PIF1/spaQ	(Zhu et al., 2015)
PIF1-HA/TAP-SPA1	(Zhu et al., 2015)
PIF1-HA	(Zhu et al., 2015)
TAP-SPA1	(Chen et al., 2010)

SPA1 #1/spaQ	This study
SPA1 #2/spaQ	This study
mSPA1 #1/spaQ	This study
mSPA1 #2/spaQ	This study
spa123/SPA1/TAP-PIF1	This study
spa123/mSPA1/TAP-PIF1	This study
phyB-GFP FL #31/phyAB	This study
phyB-GFP FL #24/phyAB	This study
phyB-GFP ΔC #44/phyAB	This study
phyB-GFP ΔC #27/phyAB	This study
phyA-211	(Nagatani et al., 1993)
phyA-211/phyB-28	This study
