# Supplementary material for: A phyB-PIF1-SPA1 kinase regulatory complex promotes photomorphogenesis in Arabidopsis
Source: Nat Commun. 2019 Sep 16;10:4216. doi: 10.1038/s41467-019-12110-y (PMC6746701; doi:10.1038/s41467-019-12110-y)
Supplement: Supplementary file 3 — Description of Additional Supplementary Files [file 41467_2019_12110_MOESM3_ESM.pdf]

Description of Additional Supplementary Files

File Name: Supplementary Data 1
Description: List of genes significantly regulated in different genotypes under red vs dark conditions.

File Name: Supplementary Data 2
Description: List of 1861 genes that are not significantly regulated in cop1-4 and spaQ but in wild-type under red light.

File Name: Supplementary Data 3
Description: List of the genes significantly regulated in spaQ but not in cop1-4 under red light.

File Name: Supplementary Data 4
Description: GO Enrichment analysis of genes overlapped in phyB FL and phyB ΔC mutant.

File Name: Supplementary Data 5
Description: GO Enrichment analysis of 561 genes that were misregulated in phyB ΔC mutant vs phyB FL.

File Name: Supplementary Data 6
Description: GO Enrichment analysis of list of genes overlapped between phyB Cterminal-dependent and SPA-dependent under red light.

File Name: Supplementary Data 7
Description: List of PIF target genes which are significantly regulated in spaQ mutants under red light.
