# Supplementary material for: A phyB-PIF1-SPA1 kinase regulatory complex promotes photomorphogenesis in Arabidopsis
Source: Nat Commun. 2019 Sep 16;10:4216. doi: 10.1038/s41467-019-12110-y (PMC6746701; doi:10.1038/s41467-019-12110-y)
Supplement: Supplementary file 11 — Reporting Summary [file 41467_2019_12110_MOESM11_ESM.pdf]

nature research

Corresponding author(s): Enamul Huq

Last updated by author(s): 08/21/2019

Reporting Summary

Nature Research wishes to improve the reproducibility of the work that we publish. This form provides structure for consistency and transparency in reporting. For further information on Nature Research policies, see Authors & Referees and the Editorial Policy Checklist .

Statistics

For all statistical analyses, confirm that the following items are present in the figure legend, table legend, main text, or Methods section.

n/a	Confirmed	
[ ]	[x]	The exact sample size (*n*) for each experimental group/condition, given as a discrete number and unit of measurement
[ ]	[x]	A statement on whether measurements were taken from distinct samples or whether the same sample was measured repeatedly
[ ]	[x]	The statistical test(s) used AND whether they are one- or two-sided<br>*Only common tests should be described solely by name; describe more complex techniques in the Methods section.*
[x]	[ ]	A description of all covariates tested
[x]	[ ]	A description of any assumptions or corrections, such as tests of normality and adjustment for multiple comparisons
[ ]	[x]	A full description of the statistical parameters including central tendency (e.g. means) or other basic estimates (e.g. regression coefficient) AND variation (e.g. standard deviation) or associated estimates of uncertainty (e.g. confidence intervals)
[ ]	[x]	For null hypothesis testing, the test statistic (e.g. *F*, *t*, *r*) with confidence intervals, effect sizes, degrees of freedom and *P* value noted<br>*Give P values as exact values whenever suitable.*
[x]	[ ]	For Bayesian analysis, information on the choice of priors and Markov chain Monte Carlo settings
[x]	[ ]	For hierarchical and complex designs, identification of the appropriate level for tests and full reporting of outcomes
[x]	[ ]	Estimates of effect sizes (e.g. Cohen's *d*, Pearson's *r*), indicating how they were calculated

Our web collection on statistics for biologists contains articles on many of the points above.

Software and code

Policy information about availability of computer code

Data collection	No software was used for data collection.
Data analysis	Following softwares were used to analyze RNA-seq data. Bowtie2 (Langmead and Salzberg, 2012), Tophat (Trapnell et al., 2012), HTseq (Anders et al., 2015), DEseq2 (Love et al., 2014), FastQC http://www.bioinformatics.babraham.ac.uk/projects/fastqc/, R www.r-project.org, For hypocotyl measurement imageJ NIH http://rsb.info.nih.gov/ij/ was used.

For manuscripts utilizing custom algorithms or software that are central to the research but not yet described in published literature, software must be made available to editors/reviewers. We strongly encourage code deposition in a community repository (e.g. GitHub). See the Nature Research guidelines for submitting code & software for further information.

Data

Policy information about availability of data

All manuscripts must include a data availability statement. This statement should provide the following information, where applicable:

- Accession codes, unique identifiers, or web links for publicly available datasets
- A list of figures that have associated raw data
- A description of any restrictions on data availability

Gene Expression Omnibus database GEO GSE112662, GEO GSE117114

Field-specific reporting

Please select the one below that is the best fit for your research. If you are not sure, read the appropriate sections before making your selection.

x

Life sciences study design

All studies must disclose on these points even when the disclosure is negative.

Sample size	Three independently harvested and dried Arabidopsis thaliana seeds were used for phenotypic analysis. Each experiment represents results from at least 50 seedlings as indicated in the method section.
Data exclusions	No data exclusions.
Replication	All experiments were replicated at least three times.
Randomization	Experimental samples were numbered and experiments were performed by investigators.
Blinding	Investigators were blinded to group allocation.

Reporting for specific materials, systems and methods

We require information from authors about some types of materials, experimental systems and methods used in many studies. Here, indicate whether each material, system or method listed is relevant to your study. If you are not sure if a list item applies to your research, read the appropriate section before selecting a response.

Materials & experimental systems

n/a	Involved in the study	
☐	☒	Antibodies
☒	☐	Eukaryotic cell lines
☒	☐	Palaeontology
☒	☐	Animals and other organisms
☒	☐	Human research participants
☒	☐	Clinical data

Methods

n/a	Involved in the study	
☒	☐	ChIP-seq
☒	☐	Flow cytometry
☒	☐	MRI-based neuroimaging

Antibodies

Antibodies used	α-PIF1 (Shen et al., 2008), α-phyB (Hirschfeld, M., et al. 1998), α-myc Sigma C3956, α-myc EMD Millipore OP10, α-HA Abcam ab9110, α-GFP Abcam ab6556, α-GFP Santa Cruz Sc-390394, α-MBP NEB E8032S, α-strep Sigma GT661, α-GST-HRP GE healthcare RPN1236, α-RPT5 Enzo lifesciences BML-PW8770-0025
Validation	Antibodies are either previously validated (references indicated) or commercially available from companies as indicated.
